# Simultaneous retrograde intrarenal surgery for ipsilateral asymptomatic renal stones in patients with ureteroscopic symptomatic ureteral stone removal

**DOI:** 10.1186/s12894-015-0016-7

**Published:** 2015-03-19

**Authors:** Dehui Lai, Meiling Chen, Yongzhong He, Xun Li

**Affiliations:** Urology Department, Fifth Affiliated Hospital, Guangzhou Medical University, 621 Gangwan Road, Huangpu District, Guangzhou, Guangdong 510700 China; Translational Medical Center, Minimally Invasive Technology and Product, Guangzhou Medical University, Guangzhou, Guangdong China

**Keywords:** Retrograde intrarenal surgery, Asymptomatic renal stone, Simultaneous treatment

## Abstract

**Background:**

Ipsilateral asymptomatic renal stone associated with symptomatic ureteral stone is not a rare event, and the recommended treatment policy was not declared clearly. This study was conducted to compare the outcomes of simultaneous retrograde intrarenal surgery (RIRS) and ureteroscopy to ureteroscopy alone for this clinical event.

**Methods:**

415 patients with symptomatic ureteral stone and ipsilateral asymptomatic renal stones were reviewed to obtain two match groups, who were treating with simultaneous modality (group A, N = 72), or ureteroscopy alone (group B, N = 72). Matching criteria were ureteral and renal stone side, duration and location, the presence of pre-stented. Perioperative and postoperative characteristics were compared between the two groups.

**Results:**

Mean stone burdens were similar between group A and B. Mean operative duration for group A and B were 72.4 ± 21.3 and 36.4 ± 10.2 min, respectively (P < 0.001). Mean hospital duration was 6.4 ± 2.9 and 5.3 ± 2.1 days in group A and B, respectively (P = 0.521). Ureteral SFR was 100% in each group. Renal SFR for RIRS was 86.1%. Complication rates in group A were higher (22.2% vs 13.9%), but the differences were not statistically significant (P = 0.358). In group A, complications were significantly less in pre-stented patients (3/25 vs 5/11, P = 0.04). Auxiliary treatment rate was significant higher in group B (69.4% vs 5.6%, P < 0.001) during follow-up (mean >18 months).

**Conclusions:**

Simultaneous RIRS for ipsilateral asymptomatic renal stones in patients with ureteroscopic symptomatic ureteral stone removal can be performed safely and effectively. It promises a high SFR with lower auxiliary treatment rate, and does not lengthen hospital duration and increase complications.

## Background

Asymptomatic renal stones are common in urological patients. They would be symptomatic without a complete retrieval at a certain time and required surgical treatment [1]. Although the current recommended method is active surveillance with an option for 2–3 years in EUA guidelines, it will be associated with a higher risk of surgical intervention [2,3]. Ipsilateral asymptomatic renal stone associated with symptomatic ureteral stone is not a rare event, and the recommended treatment policy was not declared clearly in any guidelines, especially in patients who had already removed symptomatic ureteral stone by ureteroscopy.

Retrograde intrarenal surgery (RIRS) is rapidly popular, benefited from the advance in flexible ureteroscopic instrumentation and holmium laser lithotripsy. It had been reported as an effective and definitive therapeutic option for patients with small to mid-size renal stones [4-6]. It is also recommended in some endourological centers for its high stone free rate (SFR) and low complications, when comparing with shock wave lithotripsy (SWL) and percutaneous nephrolithotomy (PCNL) in treating renal stones in size of <2 cm [4,7-9]. Although a high success rate has been showed independently for endoscopic treatment of ureteral and renal stones, there are few reports in the literature on simultaneous RIRS for asymptomatic renal stones in patients with ipsilateral ureteroscopic symptomatic ureteral stone removal.

The aim of this study was to evaluate the effectiveness and associated complications of this policy.

## Methods

We obtained approval for this study from the ethics committee of the fifth affiliated hospital of Guangzhou Medical University. Written informed consent was obtained from all the participants. This study was designed as a retrospective controlled study, approved by our hospital review board. The computerized files of 415 patients with ipsilateral symptomatic ureteral stone and asymptomatic renal stones between March 2009 and July 2013 were reviewed and a database was constructed. 72 patients who underwent simultaneous RIRS for ipsilateral asymptomatic renal stones after ureteroscopic symptomatic ureteral stone removalwas defined as group A. The matched group was 72 patients, who underwent ureteroscopic laser lithotripsy (URL) for the symptomatic ureteral stone alone (group B). Matching criteria were stone side, burden and location, as well as the presence of a pre-placed D-J stent.

Patients with congenital renal anomalies, pelvi-ureteral junction obstruction, ureteral strictures, previous SWL treatment, and urinary tract infection were excluded. Ureteral and renal stone side and location were assessed preoperatively by noncontrast spiral CT scanning. Stone burden was defined as the surface area and calculated according to the European Association of Urology guidelines. Preoperative laboratory tests included blood and urinary routine test, serum creatinine estimation, and prothrombin concentration.

### Surgical procedure

Prophlactic parenteral antibiotics were administrated in all patients. Patients were placed in the lithotomy position under continual epidural anesthesia. After retrograde pyelography, a 0.035 inch guidewire was placed in the upper tract. Ureter stones were treated using 8.0/9.8 French ureteroscope (Richard Wolf). Large stones were fragmented with holmium laser and the fragments were removed with the stone basket or grasping devices. After the ureteral stone was completely removed, a 12/14 F ureteral access sheath (UAS) (COOK) was placed with appropriate length in the patients who will undergo RIRS. The flexible ureteroscope (7.5Fr Karl Storz Flex-X, or 6/9.9Fr Richard Wolf Cobra) was inserted through the guidewire to the renal pelvis. Complete inspection of the entire collecting system was performed and small stones were removed by nitinol basket. Large stones were fragmented with holmium laser. Adequate stone fragmention was considered when fragments could remove by the stone basket or smaller than 2 mm in diameter. At the end of the procedure, the entire collecting system was inspected for the residual stones under the fluoroscopic guidance and a D-J stent was left for 4 weeks.

One month after procedure, all patients were assessed by noncontrast spiral CT to confirm the SFR. Complete stone-free was defined as the absence of any fragments. A visual analogue pain scale (VAS) was used to quantify the degree of pain. Preoperative and postoperative characteristics, complication rate, hemoglobin drop, hospital duration, SFR, auxiliary treatment rate (ATR), medical cost were compared between two groups. Auxiliary treatments were defined as the treatment for managing the residual renal stone or sever complication. Auxiliary procedure was defined as using surgical methods in the treatment during follow-up.

After the first follow-up evaluation, patients returned for an assessment with urinalysis, KUB or urinary ultrasound every 3 months during the first year and every 6 months thereafter.

Statistical analysis was done using SPSS 17.0® for Windows®. Continuous variables were compared with student *t* test and Wilcoxon test, and Univariable analysis was conducted using the Pearsonχ^2^ statistic or Fisher’s exact test for categorical data. Differences resulting in p < 0.05 were considered significant.

## Results

Patients’ demographic and preoperative characteristics were summarized in Table 1. There were no significant differences between two modalities. Perioperative and Postoperative characteristics were compared in Table 2. Mean operative duration for group A and B were 72.4 ± 21.3 (range 42.5–100) and 36.4 ± 10.2 (range 24–50) min, respectively (P < 0.001). Mean fluoroscopy time was significantly longer in group A (P < 0.001). Mean drop in the postoperative hemoglobin level was 0.5 ± 0.21 (range 0.1–0.7) g/dL in group A, which was found to be statistically significant (P < 0.001) compared with the corresponding decrease (0.2 ± 0.11, range 0.1–0.4 g/dL) in group B. However, no blood transfusion was required in both groups. VAS was higher in group A at postoperative 6 h, 12 h and 24 h, but the difference was not statistically significant at postoperative 24 h (P = 0.477). Mean hospital duration was 6.4 ± 2.9 days (range 3–12) in group A, and 5.3 ± 2.1 days (range 2–12) in group B (P = 0.521).

**Table 1:** **Demographic data of patient**

	**Group A (URL + RIRS)**	**Group B (URL alone)**	
		**(URL alone)**	
Variable	72 patients	72 patients	P
Age, year, mean (SD), range	48.2 (11.4),19–65	50.4 (13.2),22–71	0.296
Gender, no. (%)			0.468
Males	42 (58.3)	48 (66.7)	
Females	30 (41.7)	24 (33.3)	
BMI, kg/m2, mean (SD), range	22.98 (3.51),19–32	25.32 (5.12),20–31	0.084
Stone side, no. (%)			0.481
Left	30 (41.7)	36 (50)	
Right	42 (58.3)	36 (50)	
Grade of hydronephrosis, no. (%)			0.659
None	8 (11.1)	10 (13.9)	
Mild	46 (63.9)	38 (52.8)	
Moderate	18 (25)	24 (33.3)	
Ureteral stone location, no. (%)			0.405
proximal	20 (27.8)	24 (33.3)	
middle	8 (11.1)	12 (16.7)	
distal	44 (61.1)	36 (50)	
Ureteral stone burden, mm^2^, mean (SD), range	67.4 (27.2),40.2–110.3	71.3 (31),39.2–102.3	0.884
Renal stone location			0.981
Upper pole	22 (30.6)	20 (27.8)	
Middle pole	20 (27.8)	24 (33.3)	
Lower pole	30 (41.6)	28 (38.9)	
Renal stone burden, mm^2^, mean (SD), range	110.1 (42.2),68.3–170.1	124.5 (36.7),75.2–174.3	0.589
Pre-procedural placement of D-J stent, no. (%)	50 (69.5)	52 (72.2)	0.802

**Table 2:** **Perioperative and postoperative characteristics of patients**

	**Group A**	**Group B**	
	**(URL + RIRS)**	**(URL alone)**	
Variable	72 patients	72 patients	P
Operative duration, mean (SD), range (min)	72.4 (21.3),42.5–100	36.4 (10.2),24–50	<0.001
Haemoglobin drop (g/dL), mean (SD)	0.5 (0.21),0.1–0.7	0.2 (0.11),0.1–0.4	<0.001
Hospital duration, mean, (SD), range (days)	6.4 (2.9),3–12	5.3 (2.1),2–12	0.521
Complication rate, no. (%)	8 (22.2)	5 (13.9)	0.358
Modified Clavien classification			
GradeI	8 (11.1)	4 (5.6)	
Grade II	4 (5.6)	4 (5.6)	
Grade IIIa	4 (5.6)	2 (2.8)	
Pain visual analogue score (1 – 10), mean, (SD), range			
At 6 h	4.3 (1.3),4–7	3.1 (1.1),2–6	<0.001
At 12 h	2.8 (0.9),2–5	2.1 (0.7),1–4	<0.001
At 24 h	2.0 (0.3),1–4	1.8 (0.1),1–3	0.477
Ureteral stone free rate (U-SFR), no. (%)	72 (100)	72 (100)	-
Renal stone free rate (R-SFR), no. (%)	62 (86.1)	0 (0)	<0.001
Stone composition, no. (%)			0.872
Calcium oxalate	34 (47.2)	30 (41.7)	
Calcium oxalate and phosphate	20 (27.8)	24 (33.3)	
Uric acid	16 (22.2)	14 (19.4)	
Struvite	2 (2.8)	4 (5.6)	

Complication rates in group A were higher (22.2% vs 13.9%), but the differences were not statistically significant (P = 0.358). Four patients in each group were administrated by oral analgesics for post-operative pain (Clavien). Four patients had post-operative vomit (Clavien) in group A was treated by oral antiemetic. Transient post-operative fever was developed in four patients in each group and could be successfully treated with antibiotics and antipyretics (Clavien). Of group A and B, four and two, respectively, had minor ureteral perforations (Clavien a). They were successfully treated by D-J stent for 8 weeks and did not have any subsequent sequelae at follow-up. In group A, complications were significantly less in patients with pre-procedural D-J stent placement (3/25 vs 5/11, P = 0.04). Also, ureteral perforation was only encountered in patients without pre-procedural D-J placement.

One-month ureteral SFR was 100% in each group. In group A, one-month renal SFR was 86.1%. Eight failures of RIRS were due to impossible to reach the calyx containing stone. Residual fragments were seen in two patients, which were passed spontaneously during follow-up. Statistically significant was not found in stone composition between group A and B.

Follow-up data was recorded in all patients (Table 3). Mean follow-up time for all patients was 18.6 ± 9.6 months (range 12–36). The ATR was significant higher in group B (69.4% vs 5.6%, p < 0.001). In group A, two patients underwent PCNL for renal stone, while 62 auxiliary procedures were performed in 46 patients in group B, including URL (n = 10), RIRS (n = 22), ESWL (n = 26), PCNL (n = 4). Of 46 patients, Four had obstructing steinstrasse after ESWL were treated by URL, ten underwent RIRS because of significant residual stone after ESWL and two underwent PCNL for the renal stone due to failure in RIRS. Therefore, mean number of procedures per patient was significantly higher in group B (1.86 vs 1.03, p < 0.001). But mean medical cost per patient was still higher in group A (16431.2 ± 3425.3 vs 13125.1 ± 2165.4 RMB, P < 0.001).

**Table 3:** **Follow-up data of patients**

	**Group A**	**Group B**	
	**(URL + RIRS)**	**(URL alone)**	
Variable	72 patients	72 patients	P
Follow-up, mean, (SD), range (months)	18.9 (10.2),14–32	18.5 (9.4),12–34	0.675
Auxiliary treatment rate, no. (%)	4 (5.6)	50 (69.4)	<0.001
Cause of auxiliary treatment in follow-up, no. (%)			
Renal colic	2 (2.8)	10 (13.9)	
Repetatus urinary tract infection	0	6 (8.3)	
Stone induced hematuria	2 (2.8)	10 (13.9)	
Increase in creatinine levels	0	4 (5.6)	
Patient’s desire (increased stone duration)	0	20 (27.8)	
Medical expulsive treatment, no. (%)	2 (2.8)	4 (5.6)	<0.001
Patients required auxiliary procedures, no (%).	2 (2.8)	46 (63.9)	<0.001
Auxiliary procedures, no	2	62	-
URL	0	10	
FURL	0	12	
ESWL	0	26	
PCNL	2	4	
Procedure per patient, mean, (SD), range	1.03 (0.17),1–2	1.86 (0.76),1–3	<0.001
Medical cost per patient, mean, (SD), range (RMB)	16431.2 (3425.3)	13125.1 (2165.4)	<0.001
	14985.3–21325.4	9105.1–15143.2	

## Discussion

Asymptomatic renal stone associated with ipsilateral symptomatic ureteral stone is not a rare event [10,11]. URL has equivalent or superior results comparing with ESWL in treating symptomatic ureteral stone. [12] When encountering a coexisted ipsilateral asymptomatic renal stone, no established guidelines are available. Active observation, ESWL, PCNL as well as RIRS should be discussed. Previous research had showed that active observation will be associated with a higher risk of surgical intervention [2,3]. Patients choosing ESWL often needed multiple sessions to achieve higher SFR [13]. PCNL, a favoured treatment for stone >2 cm, is associated with higher potential risks, such as bleeding, urosepsis, and urine leakage [2]. Recently, simultaneously RIRS becomes feasible in treating ipsilateral renal stone and seems to be an attractive option. We compare the outcomes of this simultaneous modality to URL alone in this study.

Ureteral stone was completely removed in each group. In simultaneous RIRS group, the overall renal SFR after 1 month was 86.1%, which was similar to that of previous reports. Goldberg H et al. showed that patients with pre-procedural D-J stent can achieve a higher renal SFR (93.3% VS 71%) [14]. However, the difference was not found in this study. Inability to reach the lower pole calyx may be the main reason of RIRS failure [15,16]. We observed that eight cases were due to this. Also, the other predictive factor of renal SFR was stone size. Grasso and Ficazzola reported that RIRS can achieved an SFR of 82%, 71% and 65% with stone size of <1 cm, 1–2 cm and >2 cm, respectively [15]. RIRS may be required to clear a large stone by multiple procedures [17]. In our center, it is often performed for renal stone in size of <2 cm, which can achieved a higher SFR in one session. In this study, the simultaneous RIRS achieved 86.1% renal SFR for treating this size stone.

The other important results were lower ATR, while complications were not significantly increased. The causes of the higher ATR in URL alone group were often stone induced (Table 3). In Streem’s and Glowacki’s study, respectively, Patients with active observation, more than 70% and 48.5% required treatment due to increased stone duration or clinical symptomatic episode in next 5 years [18,19]. Although our mean follow-up period were >18 months, the ATR was 69.4% in URL alone group comparing to only 5.6% in simultaneous RIRS group. Few patients with residual stone may be one of the reasons. And the other reason was that causes of auxiliary treatment after RIRS were unexpected incidents such as complications or flexible ureteroscope damage, which is low in current reports Therefore, it is important to emphasize the possibility of auxiliary treatment in patients with URL alone is up to 70%, and with simultaneous RIRS is only required in unpredictable situation during preoperative conversation.

UAS is becoming increasingly popular worldwide because of facilitating the access, decreasing intrarenal pressure and protecting the scope [20]. However, several studies had shown that the over distention created by UAS may induce ureteral ischemia and wall injuries [21]. In this study, we found that ureteral perforations were developed in two patients in simultaneous RIRS group, who were not pre-stented. Traxer O and Thomas A reported that D-J prestenting significantly decreases the incidence of severe access sheath related injuries [22]. Moreover, overall complications were significantly less in patients with pre-procedural D-J stent (6/50 vs 10/22, P = 0.04). Thereby, it is wisdom to place DJ stent pre-procedurally in patients who were planned to undergo simultaneous RIRS.

Although RIRS had minimal invasive nature, the low morbidity was probably due to greater expertise in high-volume RIRS center. When a surgeon is still in his learning curve of RIRS, more attention should be paid in performing simultaneous modality.

Beside the invasive nature of RIRS, another disadvantage included the consumption of expensive instruments such as fragile flexible ureteroscope, nitinol basket and UAS. Large studies showed the need for repair flexible ureteroscope after an average of 18 cases [23]. Obviously, the costs for RIRS are higher than URL. In our study, although mean procedure per patient was significantly more in URL alone group, the mean medical cost per patient was still higher in simultaneous RIRS group during follow-up (mean >18 months). Simultaneous modality does not appear to be cost effective. However, SH Lee et al. reported that patients benefited from cost-effectiveness when choosing RIRS simultaneously, with respect to their health insurance system [24]. Rencently, repair for a new generation flexible ureteroscopes was needed after 20–22 procedures [25,26]. Moreover, flexible ureteroscopes can have a significantly longer lifespan (10.6 vs 21.6 uses before damage), by following guidelines and with training. [27]. Thus, we believed that the results may be changed with the developments of instruments, techniques and national health insurance system.

An interesting observation from study was that 47.8% patients in URL alone group underwent ESWL. Despite higher retreatment rates, it remains a preferred option because of non-invasive nature and high level of acceptance by patients and doctors. Although Keeley FX et al. demonstrated that ESWL for small asymptomatic renal stones does not offer any advantage to patients in terms of SFR comparing to observation (28% vs 17%, P = 0.06) [1], we found it can partly eliminate apprehensiveness of patients, and can achieve a higher SFR in upper pole renal stone. However, A policy of treating asymptomatic renal stones with ESWL may be still associated with a high risk of requiring invasive procedures > 50% patients were required additional URL, RIRS or even PCNL for obstructing steinstrasse and residual stone.

The main limitation of this study is its retrospective design. Allocation to a treatment modality depended on the surgeon’s preference. We tried to overcome this possible selection bias by comparing match groups of patients and stones. Another limitation was the small number of patients and a single center study. Therefore, a prospective randomized controlled study with a larger sample of multiple centers with a long time follow-up is needed.

## Conclusions

Simultaneous RIRS for ipsilateral asymptomatic renal stones in patients with ureteroscopic symptomatic ureteral stone removal can be performed safely and effectively.

It promises a high SFR with lower auxiliary treatment rate, and dose not lengthen hospital duration and increase complications.

Pre-procedurally Placing DJ stent in patients planned to undergo RIRS simultaneously may reduce the complication rate.

## Abbreviations

RIRS: Retrograde intrarenal surgery
URL: Ureteroscopic laser lithotripsy
ESWL: Extracorporeal shock wave lithotripsy
PCNL: Percutaneous nephrolithotomy
SFR: Stone free rate
ATR: Auxiliary treatment rate
UAS: Ureteral access sheath
VAS: Visual analogue pain scale
CT: Computed tomography

## References

[1] Keeley FX, Tilling K, Elves A, Menezes P, Wills M, Rao N (2001). Preliminary results of a randomized controlled trial of prophylactic shock wave lithotripsy for small asymptomatic renal calyceal stones. BJU Int.

[2] Turk C, Knoll T, Petrik A, Sarica K, Straub M, Seitz C (2012). Guidelines on urolithiasis.

[3] Koh LT, Ng FC, Ng KK (2012). Outcomes of long-term follow-up of patients with conservative management of asymptomatic renal calculi. BJU Int.

[4] Sabnis RB, Jagtap J, Mishra S, Desai M (2012). Treating renal calculi 1-2 cm in diameter with minipercutaneous or retrograde intrarenal surgery: a prospective comparative study. BJU Int.

[5] Sabnis RB, Ganesamoni R, Doshi A, Ganpule AP, Jagtap J, Desai MR (2013). Micropercutaneous nephrolithotomy (microperc) vs retrograde intrarenal surgery for the management of small renal calculi: a randomized controlled trial. BJU Int.

[6] Resorlu B, Unsal A, Ziypak T, Diri A, Atis G, Guven S (2013). Comparison of retrograde intrarenal surgery, shockwave lithotripsy, and percutaneous nephrolithotomy for treatment of medium-sized radiolucent renal stones. World J Urol.

[7] Ozturk U, Sener NC, Goktug HN, Nalbant I, Gucuk A, Imamoglu MA (2013). Comparison of percutaneous nephrolithotomy, shock wave lithotripsy, and retrograde intrarenal surgery for lower pole renal calculi 10-20 mm. Urol Int.

[8] Ho CC, Hafidzul J, Praveen S, Goh EH, Bong JJ, Lee BC (2010). Retrograde intrarenal surgery for renal stones smaller than 2 cm. Singapore Med J.

[9] Srisubat A, Potisat S, Lojanapiwat B, Setthawong V, Laopaiboon M (2009). Extracorporeal shock wave lithotripsy (ESWL) versus percutaneous nephrolithotomy (PCNL) or retrograde intrarenal surgery (RIRS) for kidney stones. Cochrane Database Syst Rev.

[10] Kanao K, Nakashima J, Nakagawa K, Asakura H, Miyajima A, Oya M (2006). Preoperative nomograms for predicting stone-free rate after extracorporeal shock wave lithotripsy. J Urol.

[11] Abdel-Khalek M, Sheir KZ, Mokhtar AA, Eraky I, Kenawy M, Bazeed M (2004). Prediction of success rate after extracorporeal shock-wave lithotripsy of renal stones--amultivariate analysis model. Scand J Urol Nephrol.

[12] Galvin DJ, Pearle MS (2006). The contemporary management of renal and ureteric calculi. BJU Int.

[13] Sarkissian C, Noble M, Li J, Monga M (2013). Patient decision making for asymptomatic renal calculi: balancing benefit and risk. Urology.

[14] Goldberg H, Holland R, Tal R, Lask DM, Livne PM, Lifshitz DA (2013). The impact ofretrograde intrarenal surgery for asymptomatic renal stones in patients undergoing ureteroscopy for a symptomatic ureteral stone. J Endourol.

[15] Grasso M, Ficazzola M (1999). Retrograde ureteropyeloscopy for lower pole caliceal calculi. J Urol.

[16] Resorlu B, Oguz U, Resorlu EB, Oztuna D, Unsal A (2012). The impact of pelvicaliceal anatomy on the success of retrograde intrarenal surgery in patients with lower pole renal stones. Urology.

[17] Riley JM, Stearman L, Troxel S (2009). Retrograde ureteroscopy for renal stones larger than 2.5 cm. J Endourol.

[18] Streem SB, Yost A, Mascha E (1996). Clinical implications of clinically insignificant store fragments after extracorporeal shock wave lithotripsy. J Urol.

[19] Glowacki LS, Beecroft ML, Cook RJ, Pahl D, Churchill DN (1992). The natural history of asymptomatic urolithiasis. J Urol.

[20] Stern JM, Yiee J, Park S (2007). Safety and efficacy of ureteral access sheaths. J Endourol.

[21] Lallas CD, Auge BK, Raj GV, Santa-Cruz R, Madden JF, Preminger GM (2002). Laser Doppler flowmetric determination of ureteral blood flow after ureteral access sheath placement. J Endourol.

[22] Traxer O, Thomas A (2013). Prospective evaluation and classification of ureteral wall injuries resulting from insertion of a ureteral access sheath during retrograde intrarenal surgery. J Urol.

[23] Monga M, Best S, Venkatesh R, Ames C, Lee C, Kuskowski M (2006). Durability of flexible ureteroscopes: a randomized, prospective study. J Urol.

[24] Lee SH, Kim TH, Myung SC, Moon YT, Kim KD, Kim JH (2013). Effectiveness of flexible ureteroscopic stone removal for treating ureteral and ipsilateral renal stones: a single-center experience. Korean J Urol.

[25] Binbay M, Yuruk E, Akman T, Ozgor F, Seyrek M, Ozkuvanci U (2010). Is there a difference in outcomes between digital and fiberoptic flexible ureterorenoscopy procedures?. J Endourol.

[26] Multescu R, Geavlete B, Georgescu D, Geavlete P (2010). Conventional fiberoptic flexible ureteroscope versus fourth generation digital flexible ureteroscope: a critical comparison. J Endourol.

[27] Karaolides T, Bach C, Kachrilas S, Goyal A, Masood J, Buchholz N (2013). Improving the durability of digital flexible ureteroscopes. Urology.

